# The relationship between Chinese vocational college students' adverse childhood experience and meaning in life: the mediating role of self-esteem and the moderating role of humorous coping

**DOI:** 10.3389/fpsyg.2025.1467780

**Published:** 2025-04-16

**Authors:** Ai Yun, Yi Cai, Yuanyan Hu

**Affiliations:** ^1^Mental Health Education and Counseling Center, Chongqing Chemical Industry Vocational College, Chongqing, China; ^2^Laboratory of Emotion and Mental Health, Chongqing University of Arts and Sciences, Chongqing, China; ^3^Department of Psychology, School of Education, Soochow University, Suzhou, China; ^4^Postdoctoral Workstation of Art Theory, Southeast University, Nanjing, China

**Keywords:** adverse childhood experience, the meaning in life, self-esteem, humorous coping, vocational college students

## Abstract

This study examined the relationship between adverse childhood experiences (ACEs) and meaning in life among Chinese vocational college students, along with the mediating role of self-esteem and the moderating role of humorous coping. A total of 451 students were assessed utilizing the Adverse Childhood Experiences Scale (ACES), the Meaning in Life Questionnaire (MLQ), the Rosenberg's Self-Esteem Scale (RSES), and the Coping Humor Scale (CHS). Results indicated that: (1) adverse childhood experiences significantly and negatively predicted meaning in life; (2) self-esteem partially mediated the relationship between adverse childhood experiences and meaning in life; (3) humorous coping moderated the first stage of the mediation model, specifically buffering the negative effect of adverse childhood experiences on self-esteem. In summary, adverse childhood experiences reduce meaning in life by impairing self-esteem, whereas enhancing humorous coping abilities can buffer their detrimental effects on self-esteem among college students.

## 1 Introduction

With the increasing emphasis on vocational education by the Chinese government, the number of students in higher vocational institutions has been steadily rising. As of December 2022, there were 16.9377 million students enrolled in higher vocational education in China, an increase of 907,400 from the previous year (Ministry of Education of the People's Republic of China, 2023). However, previous research has focused more on the mental health of students in regular undergraduate institutions. Vocational college students often face issues such as low academic expectations, low self-evaluation, low campus satisfaction, and poor employment prospects (Wang and Yu, 2016), making them more prone to mental health problems like inferiority, anxiety, and confusion (Zeng, 2019).

Meaning in life is an important protective factor for mental health, positively contributing to alleviating negative emotions such as depression and anxiety (Baquero-Tomás et al., 2023). Enhancing meaning in life is beneficial for improving individual mental health. Therefore, exploring ways to enhance meaning in life is of great significance for promoting the mental health of students in higher vocational institutions.

Previous studies have shown that meaning in life is influenced by various factors such as childhood experiences (Rose et al., 2023) and self-esteem (Liu et al., 2018). For example, individuals with more adverse childhood experiences are more likely to have non-suicidal self-injury behaviors, suicidal ideation, and mental health problems (Isohookana et al., 2013), which in turn lead to a lack or even loss of meaning in life (Chen et al., 2022). Additionally, studies have shown that college students with high self-esteem are more likely to experience positive emotions and have a higher meaning in life (Zhang et al., 2015). Moreover, adverse childhood experiences are closely related to self-esteem, with childhood abuse and neglect significantly negatively impacting self-esteem levels (Wang et al., 2022). These studies have respectively explored the relationships between adverse childhood experiences, self-esteem, and meaning in life but have not delved into the internal mechanisms combining these three factors. Therefore, this study aims to investigate the formation mechanisms of meaning in life in Chinese vocational college students, hoping to find effective ways to enhance meaning in life, and provide a reference for improving the mental health of students in higher vocational institutions.

## 2 Literature review

### 2.1 Adverse childhood experiences and meaning in life

Adverse childhood experiences, also known as childhood trauma or childhood neglect, refer to traumatic events experienced by individuals under the age of 18, including physical and emotional abuse. Approximately 50% of college students have experienced at least one form of childhood trauma (Hou et al., 2022). These childhood traumas are closely related to individual mental health (Nöthling et al., 2020; Tzouvara et al., 2023). They not only affect the physical and mental development of individuals during adolescence but also impair their social functions, such as learning, work, and daily life in adulthood (Choi et al., 2018). Studies have shown that individuals who have experienced multiple types of adverse childhood experiences tend to have persistent adaptation problems (Perez et al., 2016). For instance, college students with adverse childhood experiences are more likely to engage in self-harm and exhibit behaviors that devalue their self-worth (Jakubowski et al., 2018). Furthermore, individuals with more adverse childhood experiences have a lower ability to control negative emotions (Zhang S. J. et al., 2023). Additionally, adverse childhood experiences are significant factors affecting an individual's meaning in life (Jing and Ding, 2024). However, research on the internal mechanisms by which adverse childhood experiences impact meaning in life is limited and warrants further exploration.

Meaning in life refers to an integrated understanding of the value of one's existence and the significance and coherence of one's life and existence (Steger, 2012). The meaning management model suggests that although meaning in life is relatively stable, it can change due to personal experiences and shifts in values (Chang et al., 2021). For individuals who have experienced adverse childhood experiences, their sense of self-worth is often threatened, which in turn affects their self-identity and meaning in life (Chen et al., 2022). Furthermore, research has found that adverse childhood experiences are positively correlated with negative emotions. Early experiences of panic, helplessness, and other negative emotions can significantly negatively predict the level of an individual's meaning in life (Sun et al., 2023). Based on this, the first hypothesis of this study is proposed: adverse childhood experiences can negatively predict an individual's meaning in life.

### 2.2 The mediating role of self-esteem

Self-esteem refers to an individual's perception or attitude toward their own worth and is a core component of the self-system, playing a protective role in personal development (Tian, 2006). On one hand, experiences of childhood abuse and neglect can lead to low self-esteem (Li et al., 2024). According to the hopelessness-self-esteem theory, individuals are more likely to experience hopelessness and low self-worth after negative life events, which in turn can lead to enduring issues such as depression and anxiety (Ying, 1990). Therefore, positive childhood experiences are necessary for adapting to future social life and maintaining mental and physical health, as well as for reducing negative psychological imprints and enhancing self-esteem. On the other hand, the hierarchy of needs theory posits that higher-level needs can only emerge after lower-level needs are satisfied. Specifically, only when an individual's need for self-esteem is met can they pursue self-actualization, achieve peak experiences, and gain a meaning in life (Kenrick et al., 2010). Additionally, research has shown that self-esteem levels can significantly predict an individual's meaning in life, with higher self-esteem individuals experiencing greater happiness and life meaning (Zhang et al., 2015). Based on this, the second hypothesis of this study is proposed: self-esteem mediates the relationship between adverse childhood experiences and meaning in life.

### 2.3 The moderating role of humorous coping

Humor coping refers to an adaptive strategy through which individuals perceive or express humor in specific situations (Simione and Gnagnarella, 2023). It serves both as a strategy to resolve internal conflicts and as a vital means to enhance individual self-esteem (Leist and Müller, 2013).

**Figure 1:**
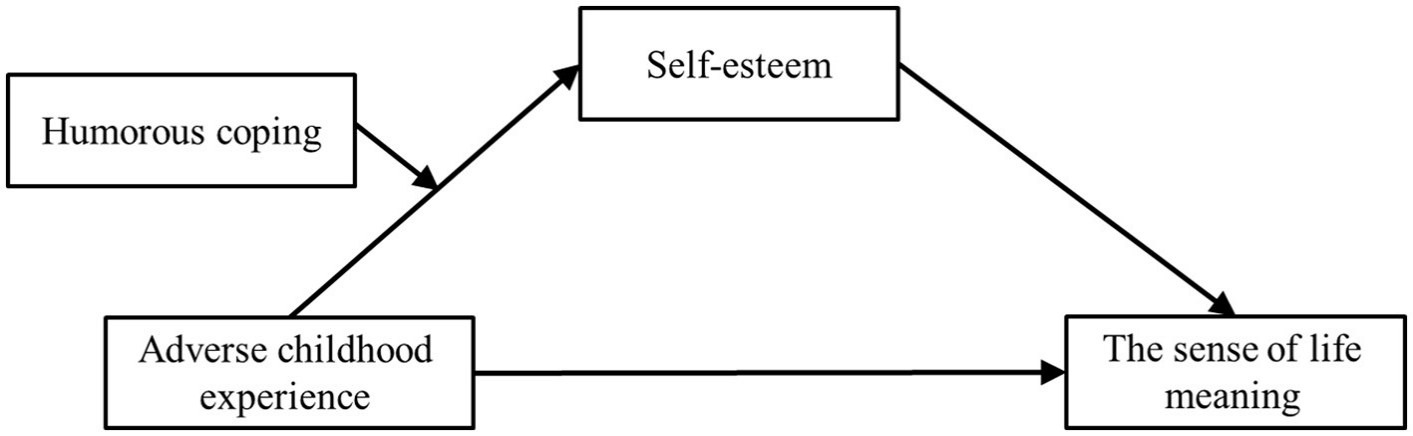
The proposed hypothesis model.

Research has shown that individuals who employ humorous coping strategies tend to have higher levels of self-esteem (Stieger et al., 2011). According to social cognitive theory, humorous coping acts as a social lubricant, alleviating anxiety and stress and helping individuals avoid being controlled by negative emotions during psychological distress, thereby enabling them to recognize their personal value (Koch, 2018). When individuals experience adverse childhood experiences that damage their self-esteem, humorous coping—as a higher-level defense mechanism—can actively integrate and effectively release negative psychological pressure, thereby enhancing their engagement with real life and affirmation of self-worth (Wellenzohn et al., 2016). Additionally, resilience frameworks suggest that when facing adversity, individuals can leverage internal or external resources to exhibit adaptive responses that protect their mental health. By employing humor as an adaptive coping strategy, individuals may enhance their emotional regulation abilities and psychological resilience. This process facilitates the restructuring of negative cognitions associated with adverse childhood experiences, thereby mitigating the negative impact of such experiences on self-esteem (Bonanno, 2008). Based on this, the third hypothesis of this study is proposed: humorous coping moderates the mediating effect of adverse childhood experiences on meaning in life, as illustrated in the theoretical model (Figure 1).

## 3 Methods

### 3.1 Participants

The study employed convenience sampling by selecting one vocational college from each of the three provinces in China: Liaoning, Heilongjiang, and Jiangsu. Data were collected through cluster-based group administration by class using electronic questionnaires. A total of 478 students participated in the survey. After excluding incomplete and insincere responses, 451 valid questionnaires were retained, yielding a 94.4% validity rate. All participants were non-psychology majors with no reported history of mental disorders. Their ages ranged from 19 to 26 years, with a mean age of 21.96 ± 2.23 years. Demographic details are presented in Table 1.

**Table 1:** Information of the participants.

	**Gender**		**Grade**				**Graduate student**
	**Male**	**Female**	**Freshman**	**Sophomore**	**Junior**	**Senior**	
***n***	193	258	80	85	78	45	163

### 3.2 Procedure and measures

First, descriptive statistics, correlation analyses, and common method bias (CMB) tests were performed using SPSS 18.0. Subsequently, the hypothesized mediation-moderation model was examined via Model 7 and Model 14 of the PROCESS macro. To assess the significance of indirect and conditional effects, the Bootstrap method with 5,000 resamples was applied to estimate 95% confidence intervals. Finally, self-esteem levels were compared between groups with high vs. low humorous coping (based on a median split) to clarify its moderating role in the model.

#### 3.2.1 The adverse childhood experiences scale

The Adverse Childhood Experiences Scale (ACES), revised by Xiao (2008), was utilized in this study. The scale captures experiences individuals had before the age of 18, including abuse, neglect, and family dysfunction, categorized into 10 types of ACEs. Each type of ACE is considered a risk factor, scoring 0 if not experienced, 1 if experienced once, with a maximum score of 10. To better understand the ACE status among vocational college students, participants were divided into low adversity group (ACE = 0–1) and high adversity group (ACE ≥ 2) (Jin et al., 2013). The Cronbach's α coefficient for this scale in our study was 0.63.

#### 3.2.2 The meaning in life questionnaire

The Meaning in Life Questionnaire (MLQ), adapted by Steger et al. (2009) and revised into Chinese by Liu and Gan (2010), consists of 5 items. Responses are rated on a 5-point scale ranging from 1 (strongly disagree) to 5 (strongly agree), with higher scores indicating stronger perceived meaning in life for the individual. In our study, the Cronbach's α coefficient for this scale was 0.76.

#### 3.2.3 The Rosenberg Self-Esteem Scale

The Rosenberg's Self-Esteem Scale (RSES), developed by Rosenberg (1965) and adapted into Chinese by Tian (2006), consists of 10 items, including 5 items that are reverse-scored. Responses are rated on a 4-point scale from 1 (strongly disagree) to 4 (strongly agree). Higher total scores indicate higher levels of self-esteem. In our study, the Cronbach's α coefficient for this scale was 0.83.

#### 3.2.4 The Coping Humor Scale

The Coping Humor Scale (CHS), revised by Yip and Martin (2006), consists of 6 items. Responses are rated on a 4-point scale from 1 (strongly disagree) to 4 (strongly agree). Higher total scores indicate a stronger tendency of humorous coping in individuals. In our study, the Cronbach's α coefficient for this scale was 0.92.

#### 3.2.5 The common method bias

The Common Method Bias (CMB) was examined using the Harman's single-factor test. Results indicated that the first factor accounted for 25.75% of the variance, which is below the critical threshold of 40% (Zhou and Long, 2004). Therefore, this study does not suffer from significant common method bias issues.

## 4 Results

### 4.1 Descriptive statistics of and correlation between the investigated variables

The results of the Pearson correlation analysis indicate that adverse childhood experiences are significantly negatively correlated with self-esteem, meaning in life, and humorous coping. Additionally, humorous coping is significantly positively correlated with both self-esteem and meaning in life (Table 2).

**Table 2:** Correlation between adverse childhood experiences, self-esteem, meaning in life, and humorous coping (*n* = 451).

**Variable**	***M***	**SD**	**1**	**2**	**3**	**4**
1. Adverse Childhood Experiences	0.86	0.35	1			
2. Self-Esteem	2.75	0.45	−0.54^**^	1		
3. Meaning in life	2.87	0.93	−0.52^**^	0.71^**^	1	
4. Humorous Coping	2.35	0.86	−0.31^**^	0.38^**^	0.78^**^	1

^**^p < 0.01.

### 4.2 Mediating role of self-esteem

A one-way ANOVA revealed no significant differences among participants from the three provinces in their scores on adverse childhood experiences, humorous coping, self-esteem, and meaning in life, *F*_(2, 448)_ = 0.82, *p* = 0.44; *F*_(2, 448)_ = 0.48, *p* = 0.62; *F*_(2, 448)_ = 0.19, *p* = 0.83; *F*_(2, 448)_ = 0.26, *p* = 0.77. The results of the independent samples *t*-test indicate that there are significant gender differences in humorous coping and meaning in life (*t* = −3.86, *df* = 449, *p* < 0.001; *t* = −4.05, *df* = 449, *p* < 0.001). Males have significantly lower levels of humorous coping and meaning in life compared to females. The one-way ANOVA results show that self-esteem varies significantly across different grades [*F*_(4, 446)_ = 25.10, *p* < 0.001]. Therefore, gender and grade will be controlled as variables in subsequent analyses.

After standardizing all variables and controlling for gender and grade, the hypothesized model was tested using PROCESS Macro Model 7. As shown in Table 3, the direct effect of adverse childhood experiences on meaning in life was significant (β = −0.76, *SE* = 0.07, *p* < 0.001, 95% bootstrap *CI* [−0.88, −0.63]). The indirect effect via self-esteem was also significant (β = −0.34, *SE* = 0.06, *p* < 0.001, 95% bootstrap *CI* [−0.47, −0.22]). Thus, self-esteem significantly mediated the relationship between adverse childhood experiences and meaning in life.

**Table 3:** Results of moderated mediation analysis (Model 7).

**Outcome variable**	**Predictor variable**	**β**	**SE**	***t***	**95%CI**	***R^2^***	***F***
MLQ	Gender	0.11	0.05	2.46^*^	[0.02, 0.20]	0.58	201.69^***^
	grade	0.25	0.02	16.60^***^	[0.22, 0.28]		
	High ACE	−0.76	0.07	−11.67^***^	[−0.88, −0.63]		
Self-esteem	Gender	0.06	0.03	1.93	[−0.01, 0.13]	0.42	63.51^***^
	Grade	0.07	0.02	4.83^***^	[0.04, 0.10]		
	High ACE	−0.27	0.07	−3.75^***^	[−0.42, −0.13]		
	Humorous Coping	0.07	0.03	2.51^*^	[0.02, 0.13]		
	High ACE × Humorous Coping	−0.51	0.09	−5.43^***^	[−0.69, −0.32]		
MLQ	Gender	0.06	0.04	1.56	[−0.02, 0.14]	0.69	252.58^***^
	Grade	0.20	0.01	14.77^***^	[0.17, 0.22]		
	High ACE	−0.34	0.06	−5.36^***^	[−0.47, −0.22]		
	Self-esteem	0.71	0.05	13.14^***^	[0.60, 0.81]		

Low adversity as the reference group. ^*^p < 0.05, ^***^p < 0.001.

To test the stability of the hypothesized model, an alternative Model 14 was examined, and the results are presented in Table 4. Compared to Model 7, Model 14 showed no significant changes in the magnitude of the indirect effect, confidence intervals, standard errors, or significance levels. Furthermore, the bootstrap confidence interval (CI) did not include zero (β = −0.25, *SE* = 0.05, *p* < 0.001, 95% bootstrap *CI* [−0.35, −0.15]). Thus, the hypothesized Model 7 demonstrates stability.

**Table 4:** Results of moderated mediation analysis (Model 14).

**Outcome variable**	**Predictor variable**	**β**	**SE**	***t***	**95%CI**	***R^2^***	***F***
Self-esteem	High ACE	−0.59	0.05	−12.14^***^	[−0.68, −0.49]	0.37	89.36^***^
	Gender	0.07	0.03	2.13^*^	[0.01, 0.14]		
	Grade	0.07	0.01	6.55^***^	[0.05, 0.10]		
MLQ	High ACE	−0.25	0.05	−4.90^***^	[−0.35, −0.15]	0.82	339.45^***^
	Self-esteem	0.67	0.05	14.23^***^	[0.57, 0.76]		
	Humorous Coping	0.44	0.03	17.38^***^	[0.39, 0.49]		
	High ACE × humorous coping	−0.02	0.06	−0.40	[−0.14, 0.10]		
	Gender	0.04	0.03	1.31	[−0.02, 0.10]		
	Grade	0.04	0.01	2.74^**^	[0.01, 0.06]		

Low adversity as the reference group. ^*^p < 0.05, ^**^p < 0.01, ^***^p < 0.001.

### 4.3 Moderated role of humorous coping

**Figure 2:**
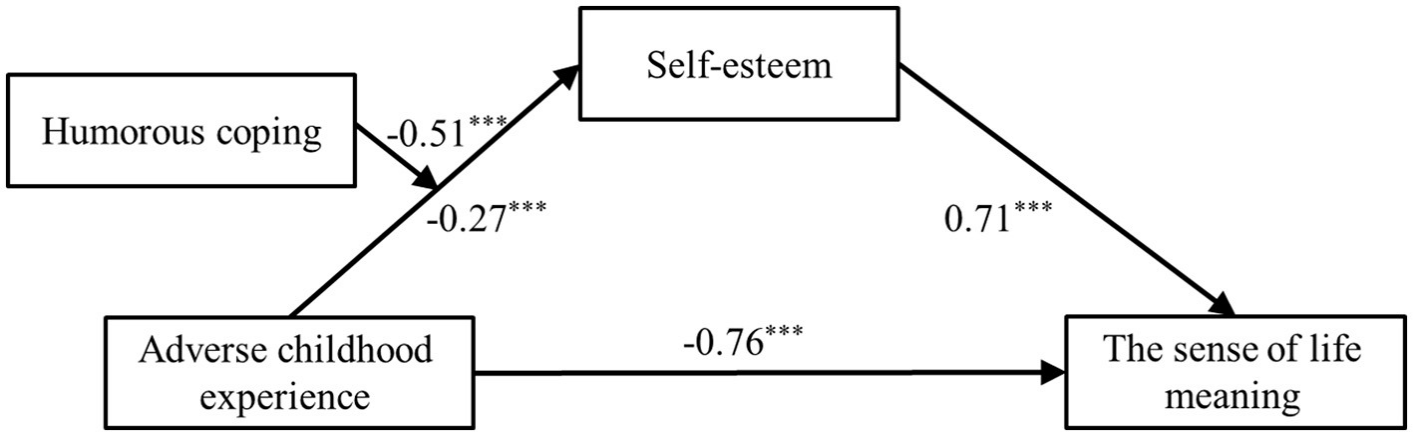
The moderated mediation model of relationship between adverse childhood experience and meaning in life. ***p < 0.001.

As shown in Figure 2, the interaction between adverse childhood experiences and humorous coping has a significant predictive effect on self-esteem (β = −0.51, *p* < 0.001), with a 95% confidence interval of (−0.69, −0.32). This indicates that humorous coping significantly moderates the relationship between adverse childhood experiences and self-esteem.

**Figure 3:**
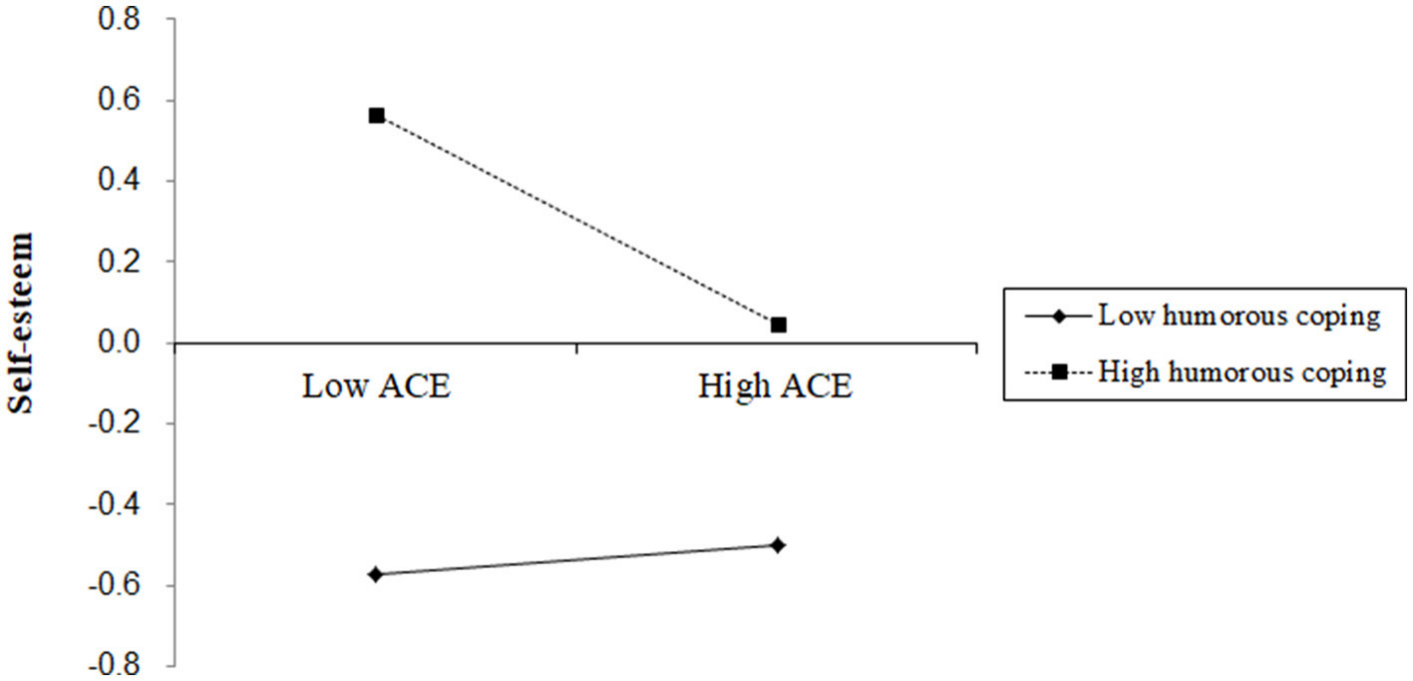
Figure of regulating effect.

To further clarify the moderating effect of humorous coping, a pairwise comparison was conducted based on the method proposed by Hayes (Hayes and Montoya, 2017). The results indicate that at low levels of humorous coping, there is no significant difference in self-esteem between the high adversity group and the low adversity group (β = −0.003, *t* = −0.024, *p* > 0.05). However, at high levels of humorous coping, there is a significant difference (β = −0.73, *t* = −12.58, *p* < 0.001), suggesting that individuals with high adversity have higher self-esteem compared to those with low adversity (Figure 3).

## 5 Discussion

This study established a moderated mediation model to examine the mediating role of self-esteem in the relationship between adverse childhood experiences and meaning in life among Chinese vocational college students. Additionally, it investigated the moderating effect of humorous coping on this mediation effect. The results indicate that adverse childhood experiences influence individuals' meaning in life through self-esteem, with humorous coping moderating the first part of this mediation effect.

First, results indicated a significant negative correlation between adverse childhood experiences and meaning in life among Chinese vocational college students. Specifically, individuals with higher adversity perceived less meaning in life compared to those with lower adversity. This finding validated Hypothesis 1 and aligned with previous research (Rose et al., 2023; Kwok et al., 2024). On one hand, from a socio-cultural perspective, traditional Chinese culture has long emphasized the ideals of “Those who excel in learning can become officials” and “All pursuits are inferior to studying,” perpetuating societal stereotypes about vocational education. Vocational college students are often labeled as “failures” destined to become blue-collar workers, exacerbating their psychological pressure (Zang, 2016). These students must not only meet academic expectations but also strive to establish existential value and future goals (Steger et al., 2008). Under such pressures, many develop poor mental health, and post-graduation phenomena like “lying flat” and “relying on parents” are increasingly observed (Jensen et al., 2022). These trends reflect a deficit in proactive and innovative engagement toward realizing life meaning among vocational students. On the other hand, childhood adversity itself directly undermines psychological resources. Experiences such as abuse and neglect may foster negative self-perceptions, leading to long-term trauma like anxiety or depression (Fanfan et al., 2023). Furthermore, family dysfunction often deprives individuals of a stable environment, eroding their trust in the world and sense of security, thereby weakening family support (Evans et al., 2013). Without adequate support, individuals struggle to cope with challenges, further diminishing their capacity to find meaning in life.

Secondly, self-esteem mediates the relationship between adverse childhood experiences and meaning in life. Increased adverse childhood experiences lead to lower levels of self-esteem, which in turn reduces individuals' subjective perceptions of their own value and life meaning. This result is consistent with previous research (Gori et al., 2022; Zhang H. et al., 2023), thus validating Hypothesis 2. On one hand, increased adverse childhood experiences may hinder the development of the central nervous system in children, causing neurological or pathological changes that increase sensitivity to negative information in adulthood, thereby lowering self-esteem (Wang et al., 2022). On the other hand, the hopelessness theory of self-esteem suggests that the acquisition of life meaning shares the same risk factors as feelings of self-esteem (Ying, 1990). That is, individuals with high adversity are more likely to experience trauma and heightened sensitivity during childhood, leading to withdrawal behaviors, and helpless cognitive styles, which negatively impact the development of self-esteem and result in a lower meaning in life (Zhou, 2016). Moreover, over time, these effects may become fixed and difficult to intervene in adulthood. Therefore, providing adequate care and positive support during childhood is crucial to avoid physical and emotional neglect, promoting healthy personality development, and enabling individuals to experience high self-esteem and a sense of value and life meaning in the future.

Finally, humorous coping moderates the mediating effect of self-esteem in the relationship between adverse childhood experiences and meaning in life. That is, humorous coping can mitigate the negative impact of adverse childhood experiences on self-esteem to some extent, thereby indirectly enhancing an individual's meaning in life. These findings not only support Hypothesis 3 but also align with prior research, suggesting that humorous coping may serve as a protective factor in alleviating the adverse effects of trauma (Sliter et al., 2014; Boerner et al., 2017). From the perspective of cognitive activity, humorous coping, as a defense mechanism, is closely related to levels of self-esteem (Marta, 2023). When individuals experience more adverse childhood events, moderate humorous coping can protect their mental health, enhance self-esteem, and help them better adapt to life. Additionally, from the perspective of social interactions, humorous coping is an effective means of communication. For individuals with high adversity in childhood, moderate humorous coping can alleviate internal anxiety and fear, thereby reducing interpersonal stress (Simione and Gnagnarella, 2023), maintaining good relationships, and enhancing self-esteem.

Based on the above findings, we recommend that Chinese vocational colleges enhance a three-tier early warning system encompassing class-level, department-level, and university-level mental health centers, with priority given to students reporting adverse childhood experiences (ACEs). Targeted psychological interventions should be provided to help these students rebuild core beliefs and assumptions about the world and themselves, thereby strengthening their sense of meaning in life. Additionally, vocational institutions could conduct regular group counseling sessions for students with ACEs, equipping them with skills to employ humorous as a coping strategy when facing stress and setbacks. To foster a supportive campus culture, colleges may leverage educational posters and social media platforms to promote humorous coping as a daily resilience-building tool.

## 6 Limitations

First, due to limitations in time, manpower, and resources, the scope of this study is relatively limited. Future research could enhance the external validity of the conclusions by expanding the sources and range of the sample. Second, this study only employed a cross-sectional design, which does not allow for the collection of longitudinal data. Future research could incorporate longitudinal designs to help researchers better interpret the relationships between variables. Lastly, this study found that self-esteem partially mediates the relationship between adverse childhood experiences and meaning in life, indicating the presence of other variables. For example, Dunkley et al. (2014) found that individuals with high levels of perfectionism and neuroticism tend to adopt maladaptive coping strategies (e.g., avoidance or emotional suppression), which are associated with poorer mental health and lower self-efficacy. Future research could explore the influence of other variables, such as personality traits and social support.

## Data Availability

The original contributions presented in the study are included in the article/supplementary material, further inquiries can be directed to the corresponding author.
